# Cholera: An Overview with Reference to the Syrian Outbreak

**DOI:** 10.1055/s-0043-1775762

**Published:** 2023-09-27

**Authors:** Munawar Hraib, Sara Alaidi, Sarah Jouni, Sana Saad, Mohammad Muna, Nour Alaidi, Zuheir Alshehabi

**Affiliations:** 1 Faculty of Medicine, Tishreen University, Latakia, Syria; 2 Department of Pathology, Tishreen University Hospital, Latakia, Syria

**Keywords:** cholera, Syria, outbreak, conflict, vaccine, earthquake, public health

## Abstract

Cholera is an acute type of diarrheal disease caused by intestinal infection with the toxin-producing bacteria Vibrio cholerae. The disease is still endemic in almost 69 countries, accounting for around 2.86 million cases and 95,000 deaths annually. Cholera is associated with poor infrastructure, and lack of access to sanitation and clean drinking water. The current cholera outbreak in Syria is associated with more than 10 years of conflict, which has devastated infrastructures and health services. There were 132,782 suspected cases reported between August 25, 2022 and May 20, 2023 in all 14 governorates, including 104 associated deaths. The recent earthquake in the region has complicated the situation, with an increase in cholera cases, and hindrance to a response to the disease. Climate change has driven a number of large cholera outbreaks around the world this year. The World Health Organization prequalifies three oral cholera vaccines. Cholera treatment mainly depends on rehydration, with the use of antibiotics in more severe infections. This review gives an overview of cholera bacteriology, pathogenesis, epidemiology, clinical manifestations, diagnosis, management, and prevention in light of global climate change and the ongoing outbreak in Syria, which poses a significant public health threat that requires urgent attention.

## Introduction

Cholera is an acute type of diarrheal disease that has affected human civilization over the centuries. Two theories were proposed to explain the origin of the disease, when the first documented cholera pandemic hit in 1817: the germ theory and the miasma (bad air) theory. For years, the scientific society favored the miasma theory and ignored any attempt to prove otherwise until Robert Koch discovered the microorganism nature of the disease in India in 1883 when he isolated the bacilli in culture and described it. Even before establishing the germ theory, the English physician John Snow successfully linked the disease to the poor hygienic state of the sewage and water systems when he investigated the 1854 cholera epidemic in London, which resulted in a better understanding of the routes of transmission and helped in managing the crisis effectively. Despite tremendous advancements in our understanding of the disease, large outbreaks still happen in endemic areas, usually linked to climatic or environmental changes. 1 Patterns of cholera transmission and infection differ between endemic and pandemic areas. These differences help in choosing the best prevention strategies suitable for each setting and guide the vaccination campaigns, which are currently more focused on emergency outbreaks, to lower the burden of the disease on healthcare systems around the world. 2 An approach like implementing case-area targeted interventions may help in containing outbreaks in higher risk areas. 3

## Bacteriology and Pathogenesis

Cholera is an acute, watery diarrheal disease, caused by intestinal infection with the toxin-producing bacteria Vibrio cholerae. V. cholerae is a highly motile, comma-shaped gram-negative bacteria with a single polar flagellum, belonging to the Vibrionaceae family, whose ecological niche is in salt or brackish water, often in association with zooplankton and shellfish. 2 4 It has hundreds of serogroups with pathogenic and nonpathogenic strains. Until recently, the disease was caused by only two serotypes (Inaba and Ogawa), and two biotypes (classical and El Tor, of toxigenic serogroup O1). In 1992, the O139, or Bengal serogroup strains of V. cholerae emerged as another epidemic variant. However, non-O1 and non-O139 serogroups may play a role in diarrheal illness and gastroenteritis. 4 5

Filamentous, lysogenic bacteriophage encodes the genes for cholera toxin. V. cholerae is transmitted via the fecal–oral route, is sensitive to acid, and most die in the stomach. Surviving virulent organisms can lodge in and colonize the small intestine, where they secrete “choleragen,” the potent cholera toxin. 5 6 This toxin binds to the plasma membrane of intestinal epithelial cells and releases an enzymatically active subunit that causes a rise in cycline adenosine monophosphate production, leading to massive secretion of electrolytes and water into the intestinal lumen. 2 6

## Epidemiology

Cholera is a water-borne disease responsible for many significant pandemics in human history; it was first recognized as a major health problem when it spread from India to other regions in Asia, Africa, and Europe, resulting in the first documented cholera pandemic in 1817. Following the first pandemic, six major pandemics erupted in different regions in the last 200 years. And the last one which started in 1961 in Indonesia is still ongoing affecting almost all continents. 1

Cholera falls within a spectrum consisting of two ends, epidemic or endemic. The two forms vary in symptom severity and affected groups. Epidemic cholera tends to be more severe and presents in large numbers simultaneously and across different age groups as the whole population lacks pre-existing immunity. On the other hand, in endemic areas small outbreaks reemerge seasonally, which creates an anticholera immunity that serves as a protective factor in adults, leaving only children under 5 years, who lack this immunity, at a higher risk of symptomatic illness. 2

In any case, serious efforts were made to advance sanitation systems in Europe and North America, making them cholera-free for decades. Nevertheless, the disease is still endemic in almost 69 countries and accounts for around 2.86 million cases and 95,000 deaths annually. 5 This estimate faces some limitations due to the poor surveillance systems currently in use and underreporting of cases, which can be attributed to concerns about the economic risks of such news on tourism and international trade. 7 And in conflict areas, it is even more challenging to obtain accurate and timely data on new cases.

In addition, cholera is closely linked to many sociogeographical factors that make some communities, such as war-torn countries, more vulnerable to outbreaks. The largest and most recently documented cholera crisis is the cholera outbreak in Yemen, a war-torn country, between 2016 and 2018. The conflict in Yemen resulted in a humanitarian crisis in terms of internal displacement and malnutrition, which paved the way for an outbreak of this scale to occur. Water supply infrastructures and wastewater treatment facilities also suffered devastating consequences, leaving much of the population without sustainable access to clean water.

All these factors, alongside climatic changes of dry/rainy seasons, contributed to a widespread transmission that led to almost 1.2 million cases and over 2500 deaths. 8 9 10 The poor state of pre-existing health facilities, increasing insecurity of the area, and the lack of a cholera preparedness and response plan prevented adequate eradication of the disease in the first smaller wave and led to a second and larger wave that resurfaced in the rainy season and affected more areas. 11 This urged the need for a more organized multi-sector and international response. Many insights and lessons can be derived from this crisis to deal with similar cases in the future.

## Cholera Outbreak in Syria

Since the conflict started, Syria has faced multiple infectious disease outbreaks, especially waterborne diseases like polio and cholera. In 2015, as the armed conflict in Northern Syria reached its peak, cholera began to erupt in overcrowded areas with severe water shortages and malnutrition.

Security constraints weakened the surveillance systems, including World Health Organization (WHO), national systems, and nongovernmental independent surveillance networks, and prevented laboratory confirmations that forced healthcare providers to rely on clinical diagnosis. The unnecessary insistence on laboratory confirmation led to an underestimation of the threat at the time. The international focus was overwhelmed by the refugee crises and more focused on the war trauma consequences, neglecting the public health situation, and the gradual collapse of the healthcare system. 12

**Fig. 1:**
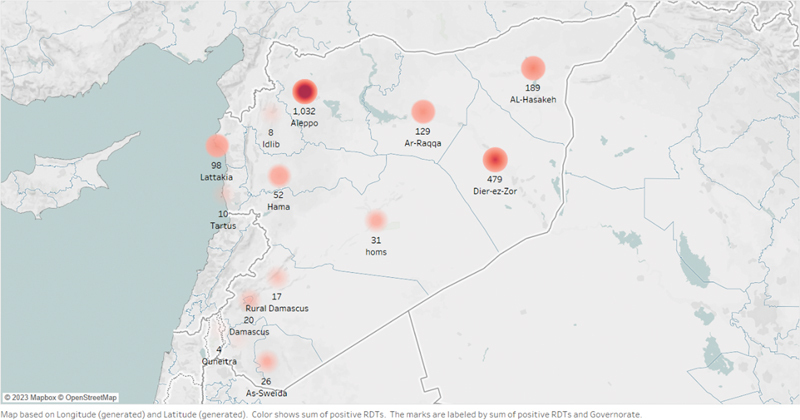
Cholera-positive rapid diagnostic tests in Syria, as of February 15, 2023.

The latest outbreak started in late August 2022. The Syrian Ministry of Health announced an outbreak of cholera on the 10th of September, in Aleppo Governorate, with 15 laboratory-confirmed cases and one fatality. In total, 132,782 suspected cases were reported in Syria between August 25, 2022 and May 20, 2023. Cases were reported in all 14 governorates including 104 associated deaths to date at a case fatality rate of 0.08%. The most affected governorates to date are Idlib (46,629 cases, 35.1%), Aleppo (38,755 cases, 29.1%), and Deir Ez-Zor (20,694 cases, 15.5%). Fig. 1 presents cholera-positive rapid diagnostic tests (RDTs), as of February 15, 2023. The main source of infection is believed to be contaminated water from the Euphrates River, which was used for drinking and irrigating nearby crops. 13 14

The dry wave that led to water shortage, combined with the disruption of sanitation systems, posed a significant risk for an outbreak in 2022. 15 The reporting and follow-up of suspected cases were found to be deficient, which decreased the reliability of the available surveillance systems and delayed an adequate response. While independent surveillance networks were previously considered more efficient and timelier, the need for more advanced and intensified systems that can function across borders and in hard-to-reach areas is more urgent than ever. 12 16

Since the first discovered case, WHO, national systems, and nongovernmental independent surveillance networks have made efforts to administer the vaccine to the affected population and establish training programs on cholera standard, its management, and prevention. 16 However, due to funding limitation, completing the rest of water sanitation and hygiene plan will not be possible soon, which leaves the door open for future outbreaks to happen especially with the lack of another accessible and affordable clean water alternative. 17

Many risk factors make Syria more vulnerable to pandemics, and make containing cholera a challenging task. Throughout more than 10 years of conflict, most of the water supply infrastructure was destroyed. Only 50% of water sanitation systems are still functioning with untreated wastewater being dumped into the environment. This left many areas of the country in a severe water shortage and a big portion of the population without any sufficient safe water source, which was exacerbated by the increasing numbers of internally displaced persons migrating from areas of conflict. Approximately 6.8 million Syrians remain internally displaced. Many of them are living in overcrowded places and unhygienic environment that increase disease transmission risk. Moreover, according to United Nation Children's Fund (UNICEF), 609,900 Syrian children are stunted. This may cause a weakening of their immune system and make them more susceptible to diseases, especially cholera. 13 18 19 20 Likewise, the ongoing conflict has led to a breakdown in the healthcare system; many healthcare facilities were put out of service and even the remaining hospitals and healthcare centers are extremely short-staffed and are not prepared to deal with large-scale pandemics, especially after the coronavirus disease 2019 crisis that exhausted the healthcare system reducing the accessibility and availability of health services throughout the country. 21

There has been debate regarding whether earthquake events are associated with cholera outbreaks. 22 However, the earthquakes that occurred on February 6, 2023, and February 20, 2023, in northwest Syria, had a significant impact on the cholera response operations, 14 led to population displacement, and may have caused sanitation infrastructure damage, and increased threats to water resources, increasing the risk of waterborne diseases including cholera. The earthquake has significantly worsened the already fragile healthcare system in the country. The direct effects of the earthquake in Northwest Syria exacerbated the spread of disease, as public health processes were paralyzed for several days and cases of cholera continued to rise. Such outbreaks shed light on the increasingly difficult humanitarian and economic crisis unfolding in Syria's cities. Cholera is an indicator of the inequalities that tear through communities. Serious and feasible actions need to be taken as soon as possible or such outbreaks will keep on emerging affecting not only Syria but nearby countries as well.

## Cholera and Climate Change

Cholera persisted to be a worldwide problem in 2022. The heavy burden of the disease, especially on developing countries in Asia and Africa, has led the World Health Organization to declare a global, multisectoral initiative to control cholera by 2030. 7

Many environmental and sociodemographic factors play a role in the transmission of the disease. The complicated way the climate interacts with infectious diseases has been studied for a decade. Cholera outbreaks exhibit seasonal patterns in endemic areas. Outbreaks were strongly linked to warmer seasons and certain geographical areas, such as tropical zones, with a series of epidemics being linked to monsoons and other extreme weather events. This association has raised questions about the impact of global warming and climate change on such a climate-sensitive disease. 23 Mean temperature, rainfall patterns, and aquatic ecosystems are the main variables that are investigated.

Cholera thrives in warm, brackish climates. Warmer temperatures and saltwater intrusion associated with sea levels rise accelerate replication rates and increase the risk of ingesting an infectious dose. In addition, this increase in mean temperature and CO2 emissions precipitate significant changes in the surface ocean layers in terms of acidity, salinity, and oxygen concentration, which also affect cholera growth. Higher replication rates lead to more intense cholera outbreaks in endemic areas and may overwhelm sanitation systems introducing the disease into previously cholera-free countries. 24 25

The change in rainfall patterns alters the dynamics of cholera transmission. Increasing rainfall leads to floods that can contaminate water sources and disrupt sanitation systems. Decreasing rainfall causes drought and sets people at severe water shortage while threatening food security. In both cases, cholera outbreaks flourish and controlling the pathogen becomes a more challenging task. 25 These climatic parameters can be implemented in predictive models that help in anticipating future outbreaks and initiating early responses. 26

## Clinical Manifestation

The incubation period of cholera ranges from 18 hours to 5 days. 2 27 After that, clinical manifestations may appear, ranging from asymptomatic to severe diarrhea. 28 The only initial symptoms can be massive painless diarrhea, up to 1 L per hour, along with watery alkaline vomiting in the severe disease. 2 27 29 30 This fluid loss can lead to severe dehydration and death if not treated. 2 29

Nevertheless, the severity of the disease could differ between endemic and epidemic countries; in endemic countries, 40 to 80% of the infections are asymptomatic. Furthermore, cholera in those areas may manifest as mild diarrhea making it hard sometimes to differentiate it from other infections. In endemic areas, severe cases tend to occur more in young children and previously unexposed individuals in those countries. 29 30

As the infection continues, the most characteristic feature of cholera is the “rice water” stool, which has a peculiar shape and fishy odor. 27 29 Abdominal discomfort and cramps caused by the expansion of fluid in the intestines have also been reported. 27 Systemic manifestations such as fever are not common and may indicate a possible secondary infection associated with cholera. Patients may suffer from intense dehydration, initially manifested by thirst and irritability, which may progress to lethargy and loss of consciousness. 2 Dehydration signs also include sunken eyes, wrinkled feet and soles, dry mouth, diminished skin turgor, rapid radial pulse, decreased blood pressure, and low urine output. In addition to Kussmaul breathing due to the acidosis resulting from losing bicarbonates in the stool or lactic acidosis related to poor perfusion. 29 Electrolyte abnormalities are a major complication of dehydration including hyponatremia, hypoglycemia, and hypocalcemia. 2 27 29 Hypoglycemia is usually more important in young children occurring due to impaired glycogen synthesis and the consumption of the glycogen stores and might cause altered consciousness, seizures, or coma. 2 28 Other complications may include acute renal failure, stroke, as well as miscarriages, and premature deliveries in pregnant patients. 2 27

## Diagnosis

Cholera should be suspected when a patient presents with a high quantity, watery, painless diarrhea, and it would be sufficient to make a clinical diagnosis if the patient also comes from or has traveled to a cholera-endemic area. 4 31 Fecal culture to isolate V. cholerae from the patient is the cornerstone for confirmation of cholera diagnosis. 2 5 Furthermore, culture can be promoted by using selective media with a high pH, which allows V. cholerae to reproduce, while preventing the growth of intestinal microflora. 2 31 The perfect culture plate for isolating cholera is thiosulfate − citrate − bile salts agar (TCBS). 31 32

In addition, V. cholerae can be diagnosed via polymerase chain reaction, which shows high sensitivity can exceed 95%, along with high specificity. 32 33 Nonetheless, many rapid diagnostic tests (RDTs) have been developed and can be employed to detect, but not to confirm, V. cholerae O1 or O130 antigen in stool samples. 2 31 32 33 However, these test performances vary significantly; some of them have high sensitivity, while their specificity are relatively low. 33 An example of RDTs is crystal VC, which is the most commonly used. 5 32 33

In Syria, the diagnosis of cholera is based exclusively on clinical diagnosis. The WHO's requirement for laboratory confirmation of cholera is unsuitable in a conflict zone such as Syria. All laboratories in northern Syria that could confirm cholera and other public health threats have been destroyed. The only laboratory in Syria is located in the capital and is not accessible to health workers caring for people in areas outside government control. 12

## Management

Without therapy, the mortality rate for severe cholera is approximately 50%. 2 27 Yet, the death rate can be reduced to less than 1% with proper treatment. 2 34

Cholera treatment begins with assessing the dehydration level of the patient and estimating the ongoing fluid losses, to determine the quantity of fluid replacement therapy to be administered to the patient. 2 The majority of patients can be managed through rapid administration of oral rehydration solution (ORS), containing electrolytes and glucose, or rice-based ORS. 2 27 32 It has been proved that a rice-based ORS is more effective than a glucose-based ORS as an appropriate treatment for cholera since it reduces the duration and volume of diarrhea. 2 27 31 32 In emergencies, we can prepare an ORS by mixing six teaspoons of sugar, and a half teaspoon of salt, with 1 L of clean water. 2 31 Breastfeeding should continue alongside the administration of ORS, except that, food should not be provided during the first 3 to 4 hours of rehydration to prevent vomiting. Thereafter, there is no need to restrict food or fluids. 27

On the other hand, patients who present with severe dehydration are at risk of shock and should be treated promptly via intravenous rehydration of nearly 350 mL/kg in the first 24 hours, preferably by Ringer's lactate solution, 27 32 to replace the whole fluid deficit at the first 3 to 4 hours. ORS then should be given instantly once the patient is capable of drinking. 2 4 Convenient antibiotics should be given to severe patients once the initial volume deficiency is treated, and vomiting has ceased, to shorten the duration of diarrhea, and reduce recovery time. 3 30 34 In addition, antibiotic therapy diminishes the excretion period of V. cholerae in the feces from 5 to 2 days or less. 27 32 35 According to the WHO, it is not recommended to mass administer antibiotics. 35

During a cholera outbreak, an antibiotic sensitivity test should be done to choose a suitable local antibiotic because cholera-endemic areas have emerged strains that showed resistance to certain antibiotics such as tetracycline-resistant strains. 27 However, there are many clinically effective antibiotics, for instance, macrolides, including azithromycin and erythromycin, or fluoroquinolones, including ciprofloxacin. 27 31 Antimotility and antiemetics have no benefit in treating cholera 4 32 In children less than 5 years old, zinc treatment is recommended, which reduces the duration and acuteness of diarrhea. 2 4 35 Furthermore, vitamin A supplementation showed reducing infant mortality. 4 Eventually, the most recurrent fault in treating cholera is underrating the fluid needs. This may occur because of errors in valuing initial dehydration and not reassessing the patient to notice the existence of the increased fluid losses. 4 For this purpose, cholera cots were developed and used to monitor stool output. 2 5

## Prevention

Developing better sanitation practices and securing safe drinking water are key roles in reducing cholera rates. However, sanitation services are still inefficient in many remote countries. In these areas, alternative behaviors can be used to reduce cholera transmission, including washing hands and drinking chlorinated water. 36 37 38 The WHO recommends vaccination during humanitarian crises as well as during outbreaks of cholera. Although vaccines are not 100% effective, they can still reduce the risk of cholera and improve health outcomes when combined with hygiene and standard cholera prevention measures. 39 40

Cholera vaccines can effectively control and prevent cholera in both short- and medium-term. 41 As in such endemic countries, a single dose of Shanchol can provide up to 40% protection against cholera for at least 6 months. 42 Several vaccines approved or prequalified by the WHO are listed and discussed in detail in Table 1 .

**Table 1:** Cholera vaccines

	Vaxchora	Dukoral	Shanchol	Euvichol-Plus
Administration	Oral 46	Oral 47	Oral 48	Oral 49
Vaccine type	Live-attenuated vaccine 46	Inactivated vaccine 46	Inactivated vaccine 47	Inactivated vaccine 49
Number of doses	One dose 46	Two doses 46	Two doses 48	Two doses 39
The age range	People between the ages of 2 and 64 35	People aged 2 years and over 39	People aged 1 year and over 48	people aged 1 year and over 39
Length of vaccine-induced immune protection	At least 3 to 6 months 35	Two years 39	Two years 48	Three years at least 39
Availability	In December 2020, the maker of Vaxchora temporarily stopped manufacturing and selling it. Which means that this vaccine is not currently available 35	Not available 39	Available for mass vaccination campaigns 39	Available for mass vaccination campaigns 39

The current global supply of oral cholera vaccine (OCV) is inadequate to fulfill all requests for two doses of preventive vaccination. Consequently, on October 20, 2022, the International Coordinating Group members (The International Federation of Red Cross (IFRC), Médecins Sans Frontières (MSF), UNICEF, and WHO) made an unprecedented decision to temporarily restrict all reactive OCV campaigns to a single dose. There is no immediate solution to augment production. While administering a single dose instead of two doses will allow more people to be protected in the short term, this approach has its limitations and it remains uncertain how long immunity will persist. 43

These shortages are not necessarily happening solely in response to the simultaneous occurrence of multiple cholera outbreaks; rather, the shortage is likely also due to global vaccine manufacturers' collective lack of interest in producing and distributing cholera vaccines, because OCV is cheap and requires bulk sales to generate profit, which is not a priority for most companies in higher-income countries. 44

## Conclusion

Cholera transmission and the patterns of cholera spread at global and regional levels remind us that behind each complex system, there is an intricate network that encodes the interactions between the system's components.

It is crucial to not only focus on outbreaks that pose a global threat and diseases that affect developed countries but also pay attention to the millions of people in poor populations who suffer from diseases with severe health, social, and economic consequences. Access to opportunities for health should not be structured by geography, commercial, or political issues.

The ongoing conflict, displacement, water insecurity, and the weakened healthcare system have contributed to the current outbreak in Syria. Urgent actions are needed to ensure the delivery of vaccines and improve access to safe water and sanitation facilities, in addition to enhancing awareness of preventive measures. The top priority should be rebuilding the healthcare system in the country and increasing access to safe drinking water and sanitation facilities, especially in conflict-affected areas. However, this will not be possible without political stability. Human health must always take precedence over political interests.
